# Supplementary material for: Elucidation of the Epitranscriptomic RNA Modification Landscape of Chikungunya Virus
Source: Viruses. 2024 Jun 12;16(6):945. doi: 10.3390/v16060945 (PMC11209572; doi:10.3390/v16060945)
Supplement: Supplementary file 1 [file viruses-16-00945-s001.zip › Supplementary table S1.pdf]

| | Reference RT (min) | $[M+H]^+$ | Product ion |
|---|---|---|---|
| **C** | 12,9 | 244,09 | 112 |
| **Y** | 11,2 | 245,08 | 155/179/209 |
| **U** | 19,7 | 245,08 | 113 |
| **m3C** | 18,2 | 258,11 | 126 |
| **m4C or m5C*** | 19,8 | | 126 |
| **Cm** | 20,6 | | 112 |
| **m1Y** | 19,7 | 259,10 | 169/193/223 |
| **m3Y** | 20,2 | | 169/193/223 |
| **m5U** | 23,0 | | 127 |
| **Um** | 26,0 | | 113 |
| **m3U** | 27,6 | | 127 |
| **s4U** | 28,7 | 261,00 | 129 |
| **A** | 32,6 | 268,10 | 136 |
| **I** | 21,2 | 269,09 | 137 |
| **hm5C** | 17,3 | 274,10 | 142 |
| **m1A** | 19,7 | 282,12 | 150 |
| **Am** | 38,5 | | 136 |
| **m2A** | 39,0 | | 150 |
| **m8A** | 40,7 | | 150 |
| **m6A** | 42,1 | | 150 |
| **Im** | 28,7 | 283,1 | 137 |
| **m1I** | | | 151 |
| **G** | 22,8 | 284,10 | 152 |
| **42C** | 30,9 | 286,10 | 112/154 |
| **m6Am** | 49,1 | 296,10 | 150 |
| **m7G** | 20 | 298,12 | 166 |
| **m1G*** | 29,5 | | 166 |
| **Gm*** | | | 152 |
| **m2G** | 30,8 | | 166 |
| **hm6A** | 35,3 | | 268/136 |
| **m27G** | 28,2 | 312,13 | 180 |
| **m22G** | 35,3 | | 180 |
| **mcm5U** | 29,2 | 317,10 | 125/153/185 |
| **m227G** | 32,9 | 326,15 | 194 |
| **mcm5s2U** | 39,6 | 333,08 | 141/169/201 |
