# Supplementary material for: Elucidation of the Epitranscriptomic RNA Modification Landscape of Chikungunya Virus
Source: Viruses. 2024 Jun 12;16(6):945. doi: 10.3390/v16060945 (PMC11209572; doi:10.3390/v16060945)
Supplement: Supplementary file 1 [file viruses-16-00945-s001.zip › Supplementary table S2.pdf]

Short name	Company	Reference
**C**	Carbosynth	NC04070
**U**	Carbosynth	NU06309
**Y**	Carbosynth	NP11297
**Cm**	Carbosynth	NM06302
**m3C**	Carbosynth	NM05757
**m4C**	Carbosynth	NM76577
**m5C**	Carbosynth	NM03720
**m1Y**	Carbosynth	NM35520
**m3U**	Carbosynth	NM06185
**m3Y**	Carbosynth	NM159403
**m5U**	Carbosynth	NM04922
**Um**	Carbosynth	NM04259
**s4U**	Carbosynth	NT06186
**A**	Carbosynth	NA01639
**I**	Carbosynth	NI06297
**Am**	Carbosynth	NM05694
**m1A**	Carbosynth	NM03697
**m2A**	Carbosynth	NM46832
**m6A**	Carbosynth	NM32281
**m8A**	Carbosynth	NM08548
**Im**	Carbosynth	NM05162
**m1I**	Carbosynth	FM163160
**G**	Carbosynth	NG06314
**m6Am**	Toronto Research Chemicals	D447415
**Gm**	Carbosynth	NM02941
**hm6A**	Toronto Research Chemicals	H805075
**m1G**	Carbosynth	NM08574
**m2G**	Carbosynth	NM35522
**m7G**	Carbosynth	NM08037
**m22G**	Carbosynth	2140-67-2
**m27G**	Carbosynth	ND45563
**mcm5U**	Carbosynth	29428-50-0
**m227G**	Carbosynth	40027-70-1
**mcm5s2U**	Carbosynth	20299-15-4
**42C**	Carbosynth	3768-18-1
**hm5C**	Carbosynth	NH35521
