# Supplementary material for: Elucidation of the Epitranscriptomic RNA Modification Landscape of Chikungunya Virus
Source: Viruses. 2024 Jun 12;16(6):945. doi: 10.3390/v16060945 (PMC11209572; doi:10.3390/v16060945)
Supplement: Supplementary file 1 [file viruses-16-00945-s001.zip › viruses-3017876-supplementary-figures.pdf]

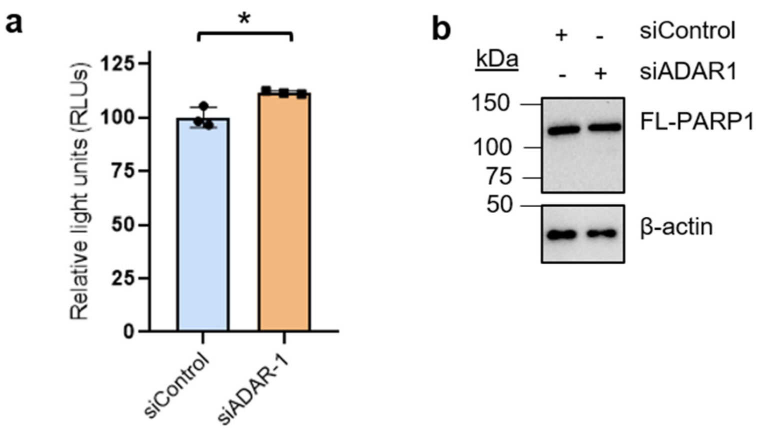

Supplementary Figure S1. Cell viability is not reduced following ADAR1 knockdown. HEK 293T cells were transfected with siControl or siADAR1. 48 h post-transfection, cell viability assays and western blots were carried out. (**a**) Cell viability assay was performed using a CellTiter-Glo Luminescent Cell Viability Assay (Promega). The bar chart shows mean values from three independent replicates with the error bars showing SD. All statistical analyses were performed using a two-tailed *t*-test. * $p < 0.05$. (**b**) Western blot analyses of full length PARP1 (FL-PARP1) (116 kDa) as well as the fragment (89 kD) of PARP1 (not detected), the latter results from caspase-3-mediated cleavage. β-actin protein is used as a loading control.

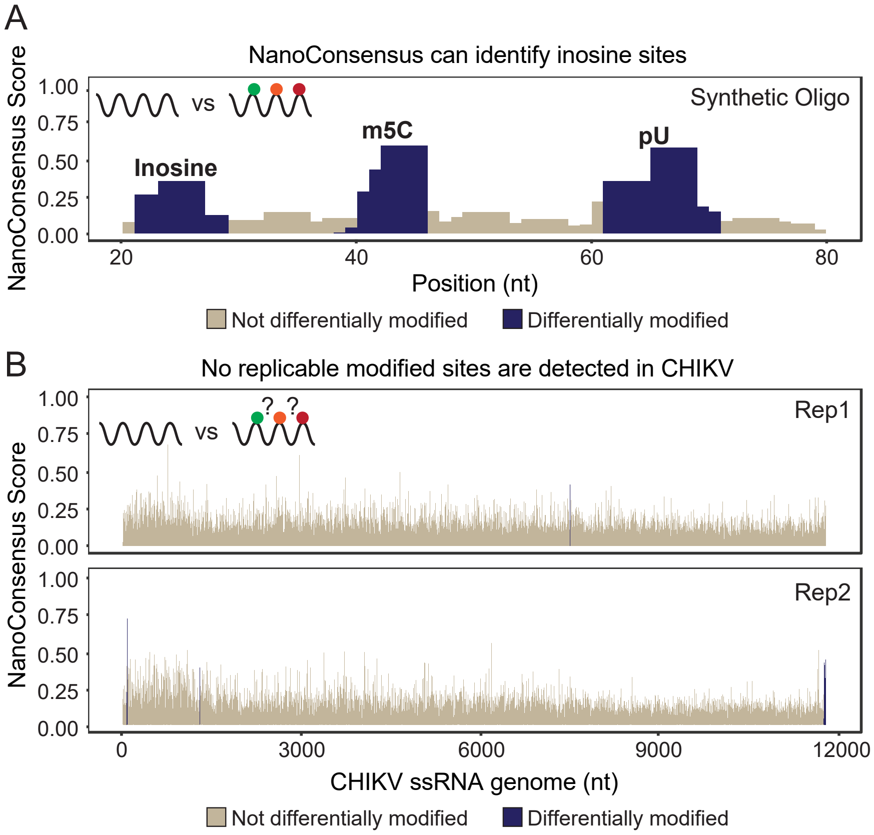

Supplementary Figure S2. No inosine sites are found in CHIKV using direct RNA sequencing (DRS). (A) NanoConsensus scores across a synthetic oligo when comparing the unmodified reads with the modified sample, which contains three modified sites: inosine, m5C and pU. In brown, non significant sites and; in blue, sites identified by NanoConsensus as differentially modified. On top of these sites, in bold, the modification found in them. (B) NanoConsensus scores along the CHIKV genome when comparing wild-type (WT) and in-vitro transcribed (IVT) datasets. In brown, non-significant sites and; in blue, sites identified by NanoConsensus as differentially modified.
